# Scientometric Analysis of Medicinal and Edible Plant *Coptis*

**DOI:** 10.3389/fphar.2021.725162

**Published:** 2021-08-12

**Authors:** Zhibang Huang, Zhengkun Hou, Fengbin Liu, Mei Zhang, Wen Hu, Shaofen Xu

**Affiliations:** ^1^ Postgraduate College, Guangzhou University of Chinese Medicine, Guangzhou, China; ^2^ Department of Gastroenterology, First Affiliated Hospital, Guangzhou University of Chinese Medicine, Guangzhou, China; ^3^ Baiyun Hospital of the First Affiliated Hospital, Guangzhou University of Chinese Medicine, Guangzhou, China; ^4^ Department of Integrative Medicine, Changsha Central Hospital, University of South China, Changsha, China; ^5^ Intensive Care Unit, Huanggang Hospital of Traditional Chinese Medicine, Huanggang, China

**Keywords:** *Coptis*, berberine, scientometric, CiteSpace, VOSviewer

## Abstract

**Objective:** A scientometric analysis to obtain knowledge mapping of *Coptis* revealed the current research situation, knowledge base and research hotspots in *Coptis* research.

**Methods:***Coptis*-related documents published from 1987 to 2020 were selected through the Web of Science Core Collection. CiteSpace, VOSviewer and Microsoft Excel were used to construct knowledge maps of the *Coptis* research field.

**Results:** A total of 367 documents and their references were analyzed. These papers were primarily published in mainland China (214), followed by Japan (57) and South Korea (52), and they each formed respective cooperation networks. The document co-citation analysis suggested that the identification of *Coptis* Salisb. species, the production of alkaloids, and the mechanisms of action of these alkaloids formed the knowledge bases in this field. A keyword analysis further revealed that the research hotspots were primarily concentrated in three fields of research involving berberine, *Coptis chinensis* Franch, and *Coptis japonica* (Thunb) Makino. Oxidative stress, rat plasma (for the determination of plasma alkaloid contents), and Alzheimer’s disease are recent research hotspots associated with *Coptis*.

**Conclusion:***Coptis* research was mainly distributed in three countries: China, Japan, and South Korea. Researchers were concerned with the identification of *Coptis* species, the production of *Coptis* alkaloids, and the efficacy and pharmacological mechanism of the constituent alkaloids. In addition, the anti-oxidative stress, pharmacokinetics, and Alzheimer’s disease treatment of *Coptis* are new hotspots in this field. This study provides a reference for *Coptis* researchers.

## Introduction

*Coptis* Salisb. is a genus of Ranunculaceae Juss., with six species found in China. Of these, the dried rhizome of *Coptis chinensis* Franch is a famous traditional Chinese medicine (TCM) and is widely used to treat diseases (Meng et al., 2018; Yang et al., 2021). It is primarily produced in Sichuan and Chongqing. *Coptis deltoidea* C. Y. Cheng et Hsiao and *Coptis teeta* Wall. are also used in TCM (Chen, Fan, and He, 2017).

From the perspective of TCM, the medicinal properties of *C. chinensis* are closely related to its efficacy. *C. chinensis* has a bitter taste and cold nature, and belongs to the heart, spleen, stomach, liver, gallbladder, and large intestine meridian. Its effects include clearing heat and dampness, purging fire and detoxification. It is often used in combination with other TCMs as a component of prescriptions with different therapeutic effects. For example, Gegen Qinlian decoction consists of four medicines: *C. chinensis*, *Scutellaria baicalensis* Georgi, *Pueraria montana* var. lobata (Willd.), and *Glycyrrhiza uralensis* Fisch. ex DC., which are used to treat patients experiencing diarrhea, dysentery and fever (Xu et al., 2015; Li et al., 2016; Lv et al., 2019). Another common prescription is the Huanglian Jiedu decoction, which is composed of *C. chinensis*, *S. baicalensis*, *Phellodendron chinense* C.K.Schneid. and *Gardenia jasminoides* J. Ellis. This prescription is used to treat symptoms such as high fever and disturbances of consciousness (Chen et al., 2017).

From a pharmacological standpoint, the rhizome of *C. chinensis* contains a variety of alkaloids (Chen et al., 2008), including berberine, coptisine, palmatine, epiberberine, jatrorrhizine, worenine, and magnoflorine, which are the main components responsible for its biological activity. Berberine is the alkaloid present in the highest concentrations in the rhizomes of *C. chinensis*. It has antipathogen (Yan et al., 2008; Mangiaterra et al., 2021), antibacterial toxin, anti-inflammatory (Cuellar et al., 2001), hypoglycemic (Lee et al., 2006), anti-gastric ulcer, antitumor, and positive muscle strength effects and exerts a negative frequency on the myocardium. With further research, an increasing number of pharmacological effects induced by berberine have been discovered, and the corresponding mechanisms of action have also been explained.

To date, many *Coptis* studies have been conducted, and some of the research results are highly influential (Shitan et al., 2003). Although many papers associated with *Coptis* have been published in journals, these studies did not examine the relationship of *Coptis* research members, the main authors, institutions or literature in the field; summarize the current research focus; or predict the future trends in the field. Therefore, considerable time is needed for beginners to systematically understand the research on *Coptis*. In addition, it is important for researchers to guide future research and improve its productivity. Thus, it is very important and necessary to overcome a series of research obstacles. Scientometric analysis is a method used to analyze the frontiers and development trends of a specific field or discipline (Hood and Wilson, 2001; Lu et al., 2020). The goal is to gain insights into the development of scientific research on a specific subject, a broader field of inquiry, and even the entire scientific knowledge system (Chen et al., 2012). This objective is achieved by mining data from scientific literature or other media on specific topics. These data are usually extracted from citation databases that generally concentrate on journal articles, conference proceedings, papers, and other types of media (Waltman, 2016). Although many papers related to *Coptis* have been published in journals, to date, there has been no research using scientometrics to analyze the knowledge base and emerging trends in *Coptis* research. Therefore, the purpose of this study is to use scientometric analysis to draw a knowledge map of *Coptis* research to outline the current background knowledge and developmental trends in this field.

## Materials and Methods

### Data Source and Search Strategy

The Web of Science (WoS) database is the most commonly used database in scientometric research (Lu et al., 2020). The “Core Collection” of the WoS database was searched as the data source on April 18, 2021, with index dates ranging from 1985 to 2020. The search strategy was “TI = (coptis OR goldthread OR goldthreads) OR AK = (coptis OR goldthread OR goldthreads)”. When we searched the WoS database, TI (title) indicated the title of a document, and AK (Author Keywords) were the keywords provided by the authors. All the records were downloaded from the WoS and imported into software for analysis. Although the value of including certain types of literature in the analysis is not high, this approach more comprehensively presents the data type distribution of the sample.

### Statistical Analysis

In this study, Microsoft Office Excel (v.2016) was used to create a figure on annual research output, and the bibliometric software VOSviewer1.6.15 and CiteSpace5.8. R1 were used to perform scientometric visualization analysis. VOSviewer was developed by van Eck and Waltman (van Eck and Waltman, 2017). This software is one of the most widely used tools in the bibliometric mapping field (Li et al., 2020). It provides visualization through similarity mapping technology and creates network visualization, in which the distance between nodes shows the relationship between them (van Eck and Waltman, 2010). In this study, the VOSviewer was used to identify the co-authors countries, author co-citations, and document co-citations. CiteSpace is a scientometric software that was developed by Professor Chaomei Chen. It was designed based on scientific revolution theory, structural hole theory, and optimal information foraging theory (Chen, 2006; Liang et al., 2017). Researchers have used CiteSpace to construct a co-authorship network and to cluster visualization of a co-occurring keyword analysis. We also created a dual-map overlay of the journals and detected the citation burst strength of the keywords. The parameters of CiteSpace are as follows: Links strength (cosine), scope (within slices). The selection criteria parameters were: g-index (k = 25), Top N (N = 50), Top N% (N = 10), link retaining factor (LRF = 3.0), look back years (LBY = 8), e for top N (e = 2.0), time span (1987–2020), and years per slice (1).

## Results

### Distribution of Publications

The distribution of publications is a key indicator that provides insights into research activities on a given topic (Zhu and Hua, 2017). This study used a descriptive analysis method to analyze *Coptis*-related publication types, annual publication trends, and published journals.

**FIGURE 1:**
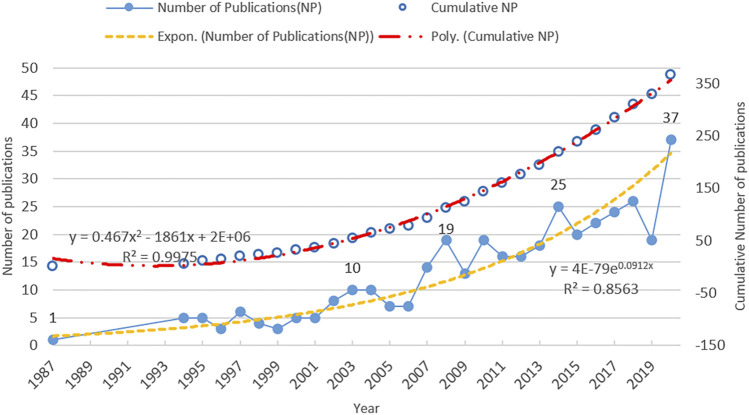
Annual distribution of *Coptis* research output.

We retrieved 367 documents related to *Coptis* from the WoS Core Collection. The types of publications retrieved included articles (319), meeting abstracts (31), reviews (10), news items (3), editorial material (2), corrections (1), letters (1) and proceedings papers (1). All the documents were published in 177 journals, of which 115 journals (65%) are members of the Committee on Publication Ethics (COPE). COPE is committed to educating and supporting editors, publishers and those involved in publication ethics to promote the culture of publishing towards one where ethical practice becomes a normal part of the publishing culture. In addition, other journals not included in COPE, also support a manuscript review process that also includes peer review. The annual distribution of *Coptis* research output is shown in Figure 1. The time interval of the publications was from 1987 to 2020, spanning 33 years. There was a total of 367 documents published during this period, with an average of 11 documents published per year, which also meant that more than one core paper was published every month. A trend line was used to fit the growth trend in the annual publication volumes. Its formula was y = 4E-79e^0.0912x^. R^2^ (0.8824), suggesting that the trend line adequately fitted the annual growth trend in publication volume. Although the annual volume of publications fluctuated, the trend line in the number of publications was growing exponentially. The figure also shows that the cumulative number of publications also had an exponential growth trend. Three significant peaks could be observed in the publication curve: 19 documents in 2008, 25 documents in 2014, and 37 documents in 2020. The number of publications began to rise sharply in 2006, each peak marked a high-yield period, and the number of records continued to increase exponentially during the later period.

### Distribution of Publication Source Journals

*Coptis*-related research papers were distributed in 177 different source journals. Supplementary Figure S1 shows the result of journal coupling analysis related to Coptis research. The distribution of journals with ≥5 publications is shown in Table 1. The top five journals, based on the number of publications, were *Planta Medica* (16), *Evidence Based Complementary and Alternative Medicine* (12), *Plant and Cell Physiology* (12), *Journal of Ethnopharmacology* (11), and *Journal of Pharmaceutical and Biomedical Analysis* (11). The output distribution of the paper sources was unbalanced, with the top five journals accounting for 16.89% of all papers. Among them, *Planta Medica*, the number one publication, accounted for 4.36% of all papers, with 385 citations and a link strength of 196.

**TABLE 1:** Journals that appeared at least 5 times in coupling analysis.

Source	Documents	Citations	Total Link Strength
Planta Medica	16	385	196
Evidence-Based Complementary and Alternative Medicine	12	54	203
Plant and Cell Physiology	12	297	279
Journal of Ethnopharmacology	11	221	193
Journal of Pharmaceutical and Biomedical Analysis	11	343	194
American Journal of Chinese Medicine	9	225	53
Fitoterapia	7	283	235
Archives of Pharmacal Research	6	251	109
Bioscience Biotechnology and Biochemistry	6	90	139
Phytochemistry	6	106	211
Journal of Biological Chemistry	5	523	272
Journal of Separation Science	5	54	172
Molecules	5	61	137
Phytotherapy Research	5	98	67

**FIGURE 2:**
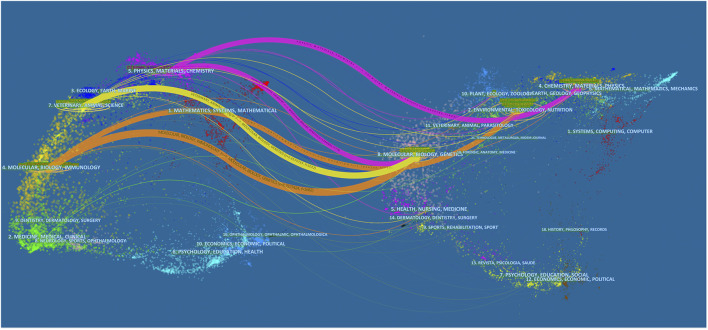
The dual-map overlay of journals.

A dual-map overlay analysis was performed on the journals. The base map is the presentation of data on different topics in the journal, and the overlay is the co-citation map of the *Coptis* research field. The dual-map overlay shows the cooperative relationship between citing publications and cited references in different fields (Hou et al., 2018) (Figure 2). Citing papers primarily centered on three fields: molecular biology and immunology, veterinary animal science, and physics and materials chemistry. Among these fields, the circles representing the fields of molecular biology and immunology and animal science were larger, indicating that the numbers of co-authors and the numbers of published publications were relatively large. The line in the figure represents the link between citing publications and cited references. Each cited field has publications citing it. Molecular biogenetics were cited by publications in other fields, suggesting that this field had an important position in the cited references of *Coptis* research.

### Network of Collaboration

#### National Collaborative Network

**FIGURE 3:**
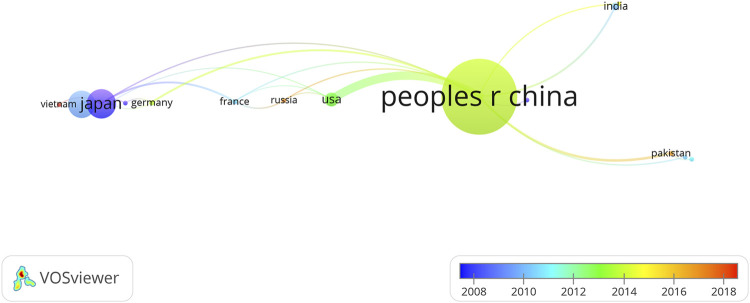
Collaboration network of countries/regions in *Coptis* research.

This section used the information at the levels of countries/regions where the research was performed to analyze the geographical distribution of documents published from 1987 to 2020. The nodes and lines in the collaborative network use different colors to represent different periods. As shown in Figure 3, the size of nodes and labels are related to the cooperative frequency of a country/region, and the thickness of the connection represents the strength of cooperation (Liao et al., 2018). The figure shows the average publication year (APY) of each country/region. Japanese and Korean publications were published relatively early, in the 2008–2012 period; American publications appeared in approximately 2013; China, Pakistan, and Russia appeared later, and they are currently countries/regions where research in the *Coptis* field is relatively active.

Table 2 lists 10 countries/regions with the highest output of *Coptis* research, corresponding to the network in the figure. Mainland China was the most prolific country in the field of *Coptis* research, with 214 papers, accounting for 58.31% of the total. China was followed by Japan (57 documents, 15.53%), and South Korea (52 documents, 14.17%). In terms of the number of citations, Table 2 shows that mainland China had the most citations (3471), followed by Japan (1710), and South Korea (1011). However, the average number of citations per document in mainland China was lower than that of Japan, South Korea, and European and American countries. This finding might be related to the publication date.

**TABLE 2:** The top 10 countries/regions in *Coptis* research.

No.	Country/Region	Continent	Documents	Citations	Total Link Strength
1	Peoples R China	Asia	214	3471	37
2	Japan	Asia	57	1710	13
3	South Korea	Asia	52	1011	6
4	United States	N. America	20	386	19
5	Taiwan	Asia	14	411	0
6	India	Asia	9	91	5
7	England	Europe	5	181	5
8	France	Europe	5	235	8
9	Germany	Europe	5	106	5
10	Pakistan	Asia	5	156	6

#### Institutional Collaborative Network

Table 3 lists the top 10 institutions that had published literature on *Coptis*. The institutional collaboration network is shown in Supplementary Figure S2. Kyoto University had published 37 papers, accounting for 10.08% of the global total, and it ranked first in *Coptis* research. It was followed by Chengdu University of Traditional Chinese Medicine (22, 5.99%) and the Chinese Academy of Sciences (17, 4.63%). In terms of citations, Kyoto University and Chengdu University of Traditional Chinese Medicine had the highest number of citations. Table 3 and Figure 3 show that Chinese universities were producing increasingly more output in *Coptis* research, and seven universities in mainland China had entered the top 10.

**TABLE 3:** The top 10 institutions in *Coptis* research.

No.	Organization	Country	Documents	%/367	Citations	Total Link Strength
1	Kyoto Univ	Japan	37	10.08	1411	21
2	Chengdu Univ Tradit Chinese Med	Peoples R China	22	5.99	284	22
3	Chinese Acad Sci	Peoples R China	17	4.63	158	36
4	China Acad Chinese Med Sci	Peoples R China	13	3.54	270	26
5	Kyung Hee Univ	South Korea	8	2.18	219	19
6	Southwest Univ	Peoples R China	8	2.18	133	6
7	Beijing Univ Chinese Med	Peoples R China	7	1.91	43	12
8	Kyungpook Natl Univ	South Korea	7	1.91	81	10
9	Wuhan Univ	Peoples R China	7	1.91	110	1
10	Zhejiang Univ	Peoples R China	7	1.91	102	9

**FIGURE 4:**
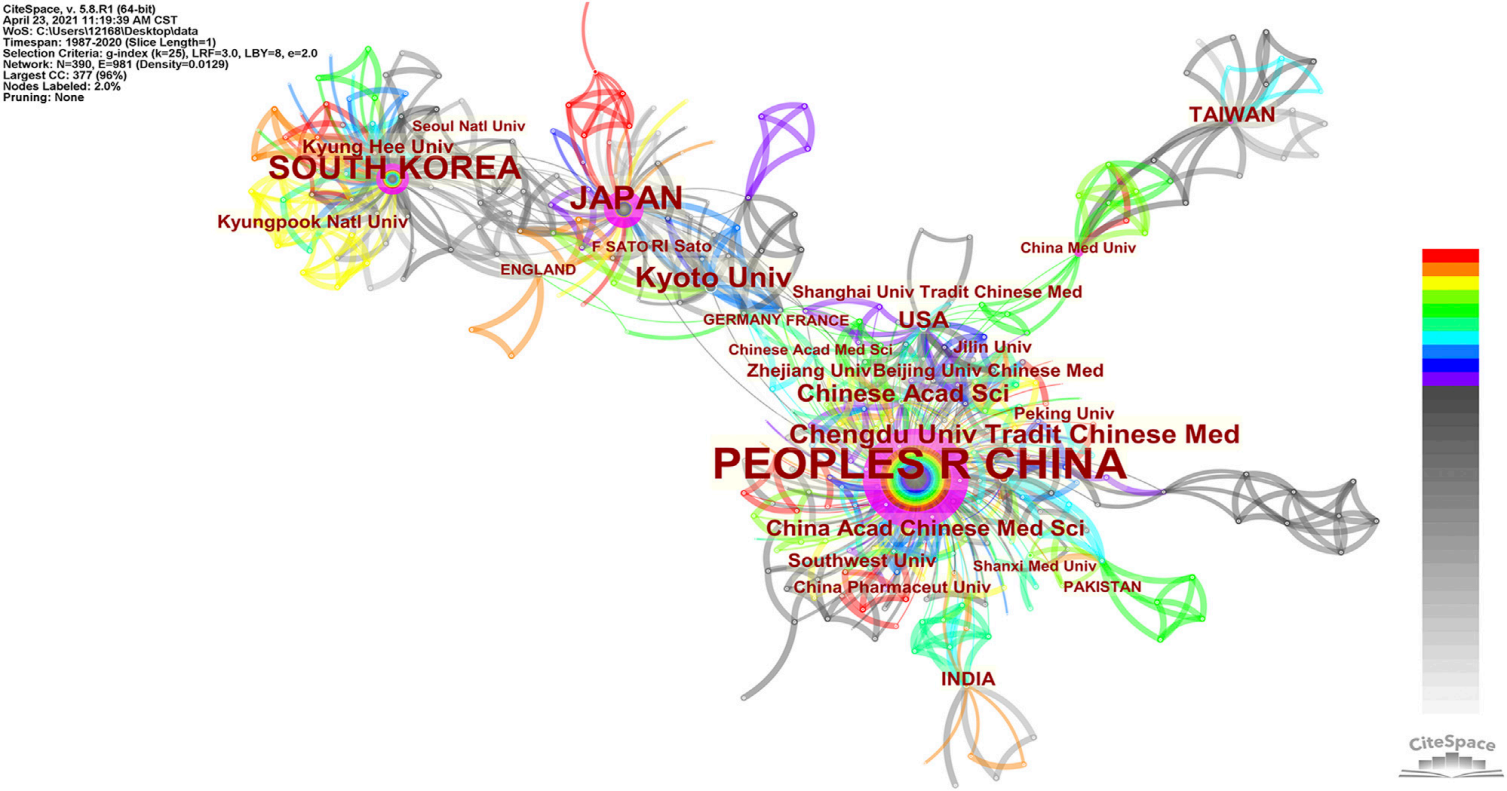
Collaboration network of countries/regions and institutions in *Coptis* research.

Figure 4 shows the cooperation network of different countries/regions and institutions over the same period, from which we could obtain the primary research institutions of each country/region and their evolution. The results showed that *Coptis* research was largely concentrated in three countries: mainland China, Japan, and South Korea. The primary research institutions in mainland China were Chengdu University of Traditional Chinese Medicine (Wu et al., 2016), Chinese Academy of Sciences, China Academy of Chinese Medical Sciences, Southwest University, and other institutions; most papers were published within the 2010–2017 period. Japanese research was primarily performed at Kyoto University (Shitan et al., 2003; Kato et al., 2007), and the publication dates were mainly during the period of 2005–2010. South Korea’s primary institutions were Kyung Hee University (Bae et al., 1998) and Kyungpook National University, and the publication dates of the literature were primarily concentrated in the year 2013.

#### Author Collaborative Network

**FIGURE 5:**
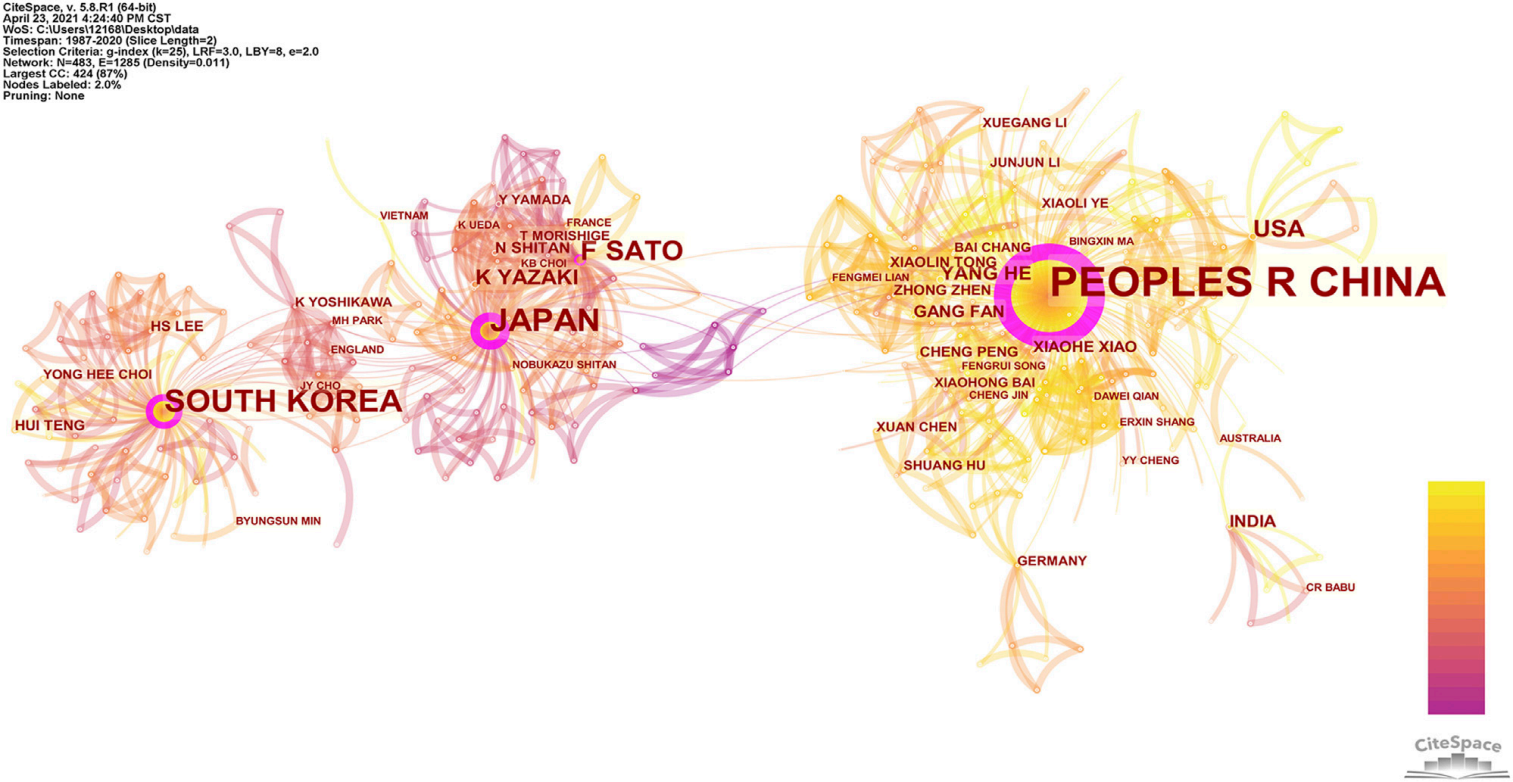
Countries/regions and authors collaboration network of *Coptis* research.

Collaborative authors form research communities, in which the collaboration between authors within a research community is stronger than the collaboration with authors outside this community (Chen, Song, et al., 2008). As shown in Figure 5, the author cooperation network in the *Coptis* research field mainly consists of three clusters, which are composed of researchers from China, Japan, and South Korea. Chinese scholars had the largest cooperation network and the largest number of scholars. The author’s APY can also be calculated to show the time dynamics of the cooperation (Hong et al., 2020). The lines between the nodes in the figure show different colors: yellow represents a recently published date, and purple represents earlier published date. In examining the figure, it was clear that Japanese and Korean scholars published studies earlier, while Chinese scholars published studies during a later time period.

There were a total of 1550 co-authors citing publications on *Coptis*-related research. Among them, 24 authors had published ≥5 papers. The author with the most published papers was Sato F with 32 papers, followed by Yazaki K (17 papers), and Shitan N (12 papers). These were all researchers engaged in *Coptis* research at Kyoto University and were primarily concentrated in the school’s Research Institute for Sustainable Humanosphere and Graduate School of Biostudies. The fourth- and fifth-ranked authors were He Y and Fan G, respectively, who were from the College of Medical Technology and State Key Lab of Characteristic Chinese Medicine Resources at Chengdu University of Traditional Chinese Medicine.

**FIGURE 6:**
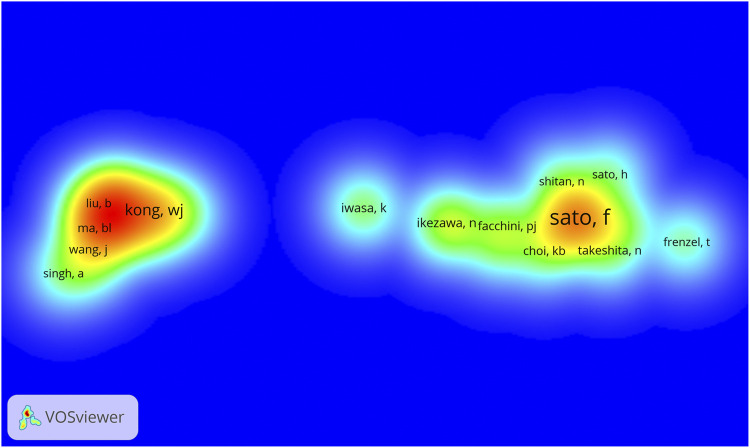
Cluster density view of the author co-citation analysis of *Coptis* research.

Author co-citation occurs when two authors are cited by other publications (Ke et al., 2020). By analyzing authors co-cited relationship, an author co-citation network can be obtained, which reveals the highly cited authors and academic communities within references in this research field (Huang et al., 2019). We extracted authors whose cited frequency was ≥15 to construct an author co-citation density view, as shown in Figure 6. In the density map, the larger the author’s label, the higher the frequency of the author’s papers being cited. The higher the frequency of author’s papers being cited, the higher density of the view, and the closer the view is to red. The figure shows that papers published by Japanese scholar Sato F at Kyoto University as the first unit had been cited 90 times in total, ranking him first among all authors. He and Morishige T (30), Ikezawa N (22), Yamada Y (20), and other major authors formed the largest network of cited references. The second largest was the subnetwork formed by the Chinese scholars Kong WJ (42) and Chen JH (37) and the Korean scholar Jung HA (41).

### Topics and Keywords

At the macro level, *Coptis* research topics can be characterized by the professional journals in which the papers are published. At the micro level, natural language processing can be used to extract terms that characterize the paper topic from the titles, abstracts, and keywords of these journal articles to analyze and research the main topic investigated (Chen and Wu, 2017); although, the author selects the appropriate keywords that offer a higher-level of generalization of the theme of the study, so an analysis of the keywords provided by the author can also characterize the core topic of the publication (Niazi and Hussain, 2011). The topic mining results of natural language processing can more clearly characterize the structure of the field, while the results analyzed by using keywords allow a more intuitive understanding of the research content of the field.

**FIGURE 7:**
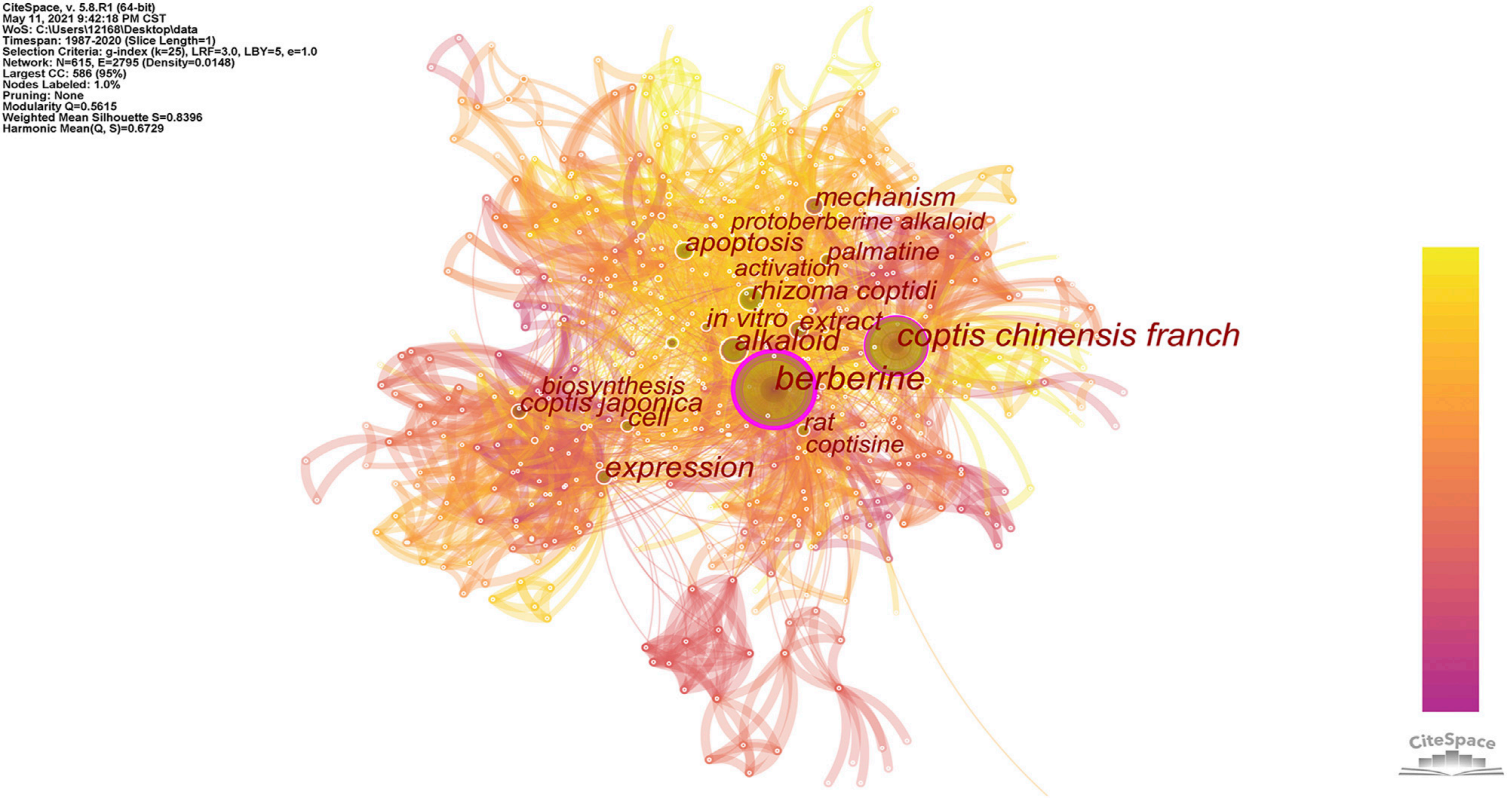
Map of keyword co-occurrence related to *Coptis* research.

Using the keyword processing method, 615 different words were identified from the keywords of 367 *Coptis*-related research documents, and high-frequency words were filtered and are presented in Figure 7. Table 4 lists the keywords with frequency ≥10 appearances. The colors of the nodes and links in the figure represent different dates, while the sizes of the nodes and labels are proportional to the word frequency. The word frequency represents the number of papers in which the keyword appears. The nodes in purple circles show high betweenness centrality, with a value of ≥0.1, which also is an indication of the importance of these nodes (Qian, 2014), which are often connected to different subnetworks and are the intermediary or bridge between nodes. On combining the graphs, we can see that berberine was the keyword with the highest frequency. It first appeared in 1994 in the keywords of the collected publications, and its betweenness centrality was 0.61, which was the highest in the keyword co-occurrence network, indicating that it played an important connecting role. The second most frequent keyword was *C. chinensis*. Its frequency was 125, and the betweenness centrality was 0.39, which suggested that *C. chinensis* received widespread attention from researchers.

**TABLE 4:** Keywords that appeared at least 10 times in *Coptis* research.

No.	Keyword	Freq	Degree	Centrality	Year	Cluster ID
1	berberine	129	197	0.61	1994	1
2	Coptis chinensis Franch	125	162	0.39	1995	2
3	alkaloid	57	96	0.14	1994	1
4	rhizoma coptidi	40	65	0.06	2005	1
5	Coptis japonica	32	57	0.09	1994	0
6	expression	31	91	0.17	1994	0
7	extract	24	70	0.1	2007	4
8	apoptosis	23	62	0.08	2005	8
9	mechanism	23	64	0.11	1996	3
10	Cell	21	67	0.09	1994	0
11	*in vitro*	19	65	0.09	1999	6
12	ranunculaceae	16	39	0.03	1994	3
13	Rat	15	43	0.07	2007	6
14	palmatine	14	43	0.04	1996	3
15	plant	13	13	0.01	1994	1
16	constituent	11	38	0.04	1995	1
17	activation	11	41	0.03	2006	4
18	identification	10	25	0.03	1997	2
19	biosynthesis	10	49	0.06	1994	0
20	protoberberine alkaloid	10	40	0.03	2006	3
21	coptisine	10	41	0.04	2000	10
22	adenosyl l methionine	10	35	0.01	1994	0

**FIGURE 8:**
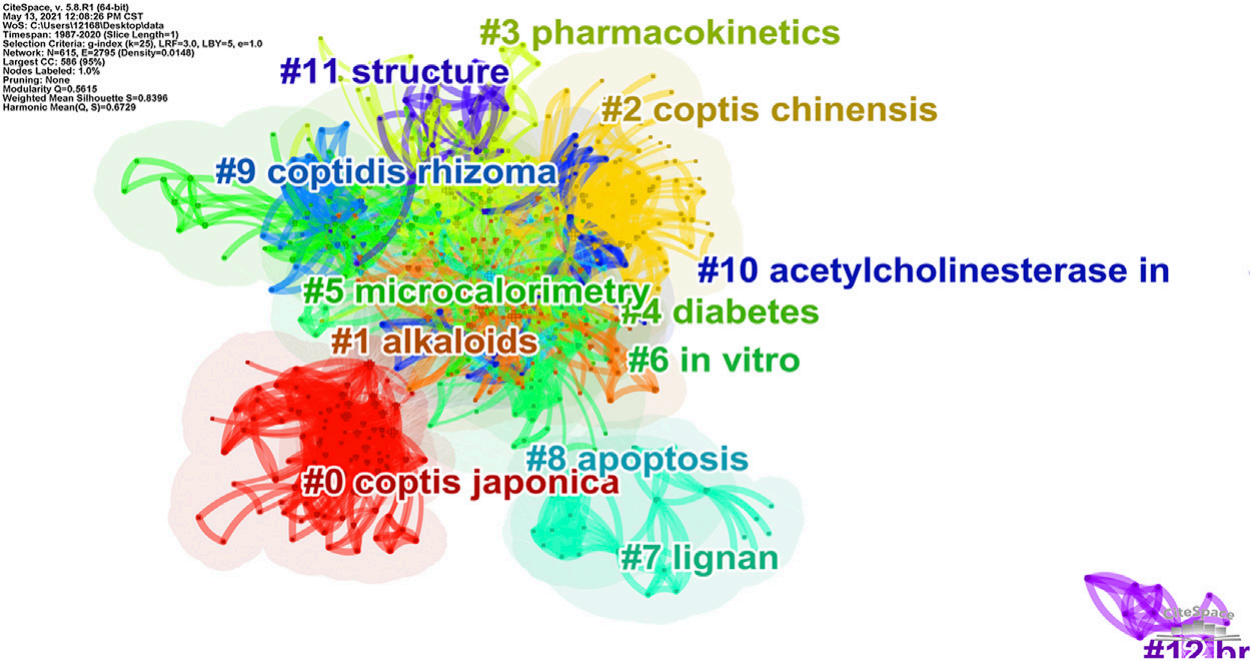
Cluster map of keyword co-occurrence in *Coptis* research.

The distance between keywords in the cluster map is an index of the similarity between them. Two keywords with similar semantics are relatively closer in the cluster graph, so the keywords are spatially clustered into categories of different sizes. In this study, the log-likelihood rate (LLR) was used to divide the obtained keyword co-occurrence network into clusters; the relevant parameters were adjusted to clarify the obtained clusters and made them more representative. After clustering, the nodes are assigned to different clusters, which are represented by different colors; the cluster map and information obtained are shown in Figure 8 and Supplementary Table S1. According to the results of the analysis, this research can be divided into 13 main research areas. The cluster with the largest size was Cluster 0 (*Coptis japonica*), followed by Cluster 1 (alkaloid), and Cluster 2 (*Coptis chinensis*). This showed that *Coptis japonica* (Thunb.) Makino occupied an important position in this field and was a hot research topic that should be explored. Alkaloids are effective components of *Coptis*. The node with the highest centrality in this cluster, berberine, is an alkaloid. *C. chinensis* is a commonly used Chinese medicine, and its primary research involved scholars from China.

**FIGURE 9:**
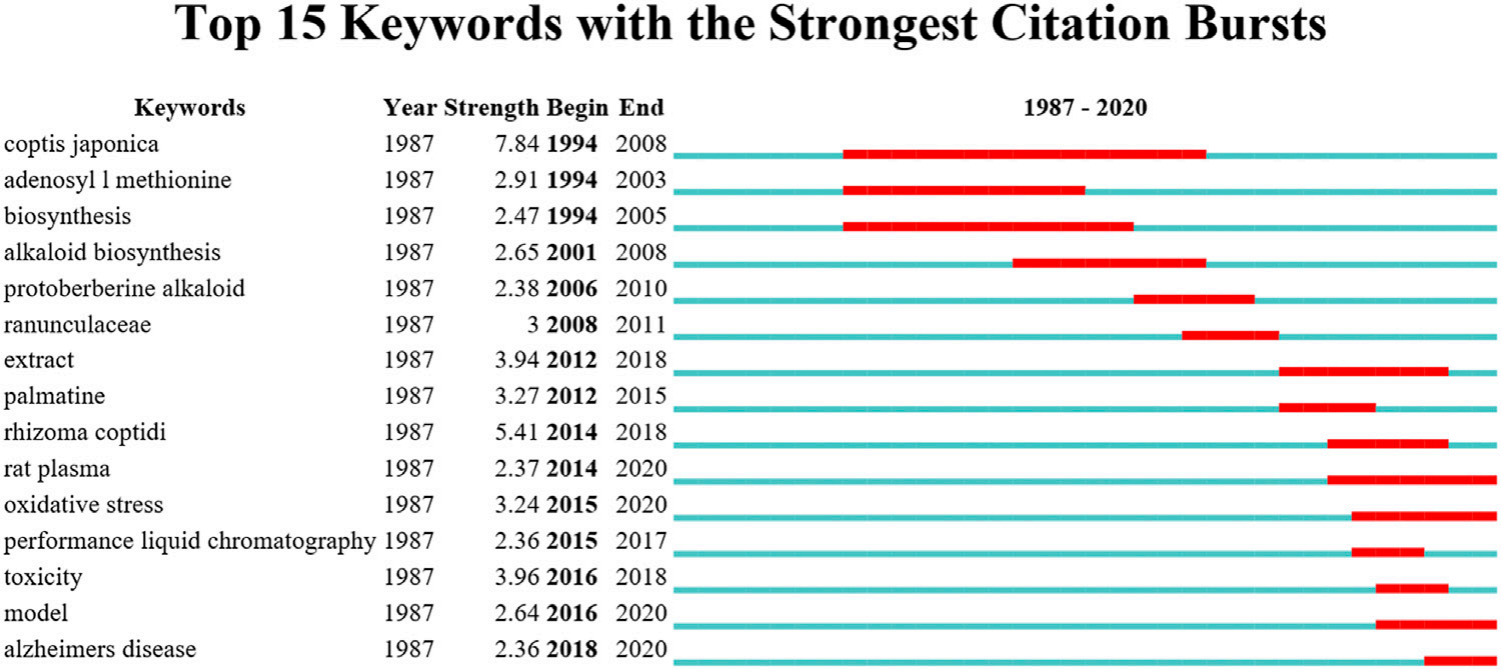
Top 15 keywords with the strongest citation bursts.

Burst keywords can show how the frequency of keyword appearance has changed in the short term. The more burst nodes a cluster contains, the more active or emerging the trends there are in this field. The top 15 keywords with the strongest citation burst results in the *Coptis* research are shown in Figure 9. From the view of burst strength, *C. japonica* was the keyword with the strongest burst strength and the longest burst period. It emerged during 1994–2008, indicating that *C. japonica* was a research hotspot in this field for over a decade. The results of class 0 were consistent. From the perspective of emergence of the keywords over time, oxidative stress, rat plasma and Alzheimer’s disease were the keywords having the most recent burst time, and these topics are new hotspots in *Coptis* research (Cao et al., 2018; Li et al., 2019).

### Document Co-citation Network

**FIGURE 10:**
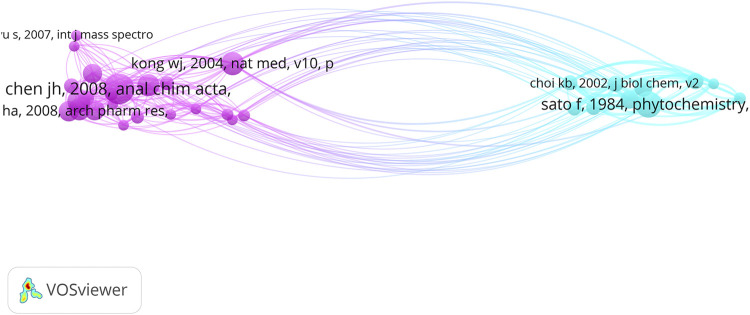
Network map of references co-citation of *Coptis* research.

This study uses the document co-citation method to analyze the relationship among cited references. We extracted 8346 documents from the references, limited the number of citations to ≥10, and obtained a co-cited network of 32 references and 233 links, as shown in Figure 10. Each node in the graph corresponds to a reference. The size of the node and the font indicates the frequency with which the reference is cited. The color of the node indicates the cluster to which the reference belongs, and the line between the nodes indicates the co-cited relationship between the references. Table 5 summarizes information relative to the top ten cited references.

**TABLE 5:** Frequently cited references in *Coptis* research.

No.	Cited Reference	Citations	Total Link Strength
1	Chen Jh, 2008, Anal Chim Acta, v613, p184, doi 10.1016/j.aca. 2008.02.060	28	25
2	Sato F, 1984, Phytochemistry, v23, p281, doi 10.1016/s0031-9422(0080318–0)	24	42
3	Kong Wj, 2004, Nat Med, v10, p1344, doi 10.1038/nm1135	21	30
4	Yuan Lj, 2006, Plant Food Hum Nutr, v61, p139, doi 10.1007/s11130-006-0023–7	21	18
5	Jung Ha, 2009, Biol Pharm Bull, v32, p1433, doi 10.1248/bpb.32.1433	20	22
6	Tang J, 2009, J Ethnopharmacol, v126, p5, doi 10.1016/j.jep. 2009.08.009	20	16
7	Jung Ha, 2008, Arch Pharm Res, v31, p1405, doi 10.1007/s12272-001-2124-z	19	20
8	Yan D, 2008, J Biochem Bioph Meth, v70, p845, doi 10.1016/j.jbbm. 2007.07.009	19	16
9	Kuo Cl, 2004, Cancer Lett, v203, p127, doi 10.1016/j.canlet. 2003.09.002	18	21
10	Morishige T, 2000, J Biol Chem, v275, p23398, doi 10.1074/jbc.m002439200	17	50

The references in the table are listed in descending order based on the number of citations. The most frequently cited reference was published by Chinese scholar Chen JH in 2008, “Analysis of alkaloids in *Coptis chinensis* Franch by accelerated solvent extraction combined with ultra-performance liquid chromatographic analysis with photodiode array and tandem mass spectrometry detections”. The second most frequent citations involved the research result of Japanese scholar Sato F published in *Phytochemistry* in 1984. This result was followed by a paper published by Chinese scholar Kong WJ in *Nature Medicine* in 2004.

## Discussion

### General Information

*Coptis* is used in the form of decoctions for complementary foods, dietary cures, and disease treatment in traditional medicine, which is homologs of medicine and food. Among the 367 documents we have obtained, six documents showed that *Coptis* can be used as an edible plant. The topic of these six studies involves the *Coptis chinensis* inflorescence, which is composed of peduncle, rachis and flowers. It is a by-product of *C. chinensis*. In the habitat of Tujia National Minority in China, the *C. chinensis* inflorescences are prepared as teas and are sold together with other functional foods in the local market (Ma et al., 2016). In addition, these studies mainly focused on the antioxidative, anti-phototoxicity, hypoglycemic, and hypocholesterolemic effects of *C. chinensis* inflorescence as an edible plant. Of course, *Coptis* is also a medicinal plant, which is the focus of most studies. *Coptis* has many functions, and exerts antipathogen, antibacterial toxin, anti-inflammatory, hypoglycemic, anti-gastric ulcer, and antitumor activity. The medicinal efficacy and pharmacological mechanism of *Coptis* are also the focus of research in this field.

The output of papers is one of the basic indicators for measuring the development of *Coptis* research, reflecting the degree of activity in the *Coptis* field. The trend line in annual publications shows that the number of papers had increased exponentially, with the largest number in 2020, reaching 37. This information showed that *Coptis* research has received attention from researchers, and an increasing number of resources (funds, scholars) will be invested in this field. *Coptis* research will become more active.

### Journals Distribution

The citing documents in this study were from 177 journals. Upon combining the number of documents and the number of citations, we found that *Planta Medica* was the journal with the most publications, and it contributes the most to *Coptis* research. The *Journal of Biological Chemistry* (JBC) was the most cited journal, and it contributed the greatest number of citations to this research. *Planta Medica* is one of the leading international journals in the fields of medicinal plants and natural product research. The journal covers the following research fields: biological and pharmacological activities, natural product chemistry and analysis research, pharmacokinetic research, natural product formulation and delivery systems. JBC mainly publishes the latest substantive scientific discoveries in various fields of biochemistry and molecular biology and clarifies the molecular and cellular basis of biological processes.

The dual-map overlay of journals indicates that the number of citing publications and coauthors in the fields of molecular biology and animal science was relatively large, indicating that *Coptis* research-related publications were mainly published in core journals in the fields of molecular biology and animal science. In cited references, articles in the molecular biology field had been cited by other areas, suggesting that this field had an important position in the cited references on *Coptis* research. This finding provided an important reference for new researchers in the *Coptis* field to perform experiments and write papers.

### Cooperation Network

*Coptis* research publications were primarily concentrated in China, Japan, and South Korea. Judging from the date of publication, studies from Japan and South Korea were published earlier, and those from China were published later. China is currently a relatively active country in *Coptis* research. In terms of the number of publications, China was the most prolific country, followed by Japan and South Korea. We believe that the reasons are as follows. First, Asia is a region where traditional medicines are used and studied more frequently. Among them, China, Japan, and South Korea are the most obvious centers for this work. Second, China is the country with the highest production and application of *Coptis chinensis*. Finally, there are more Chinese medicine researchers in China than other countries.

In the author cooperation network, the author with the most documents, Sato F, is a researcher from Kyoto University, Japan. He formed a cooperative team with many Japanese researchers, including Yazaki K and Shitan N (Shitan et al., 2013). The second was the cooperative team formed by Chinese researchers He Y and Fan G (Li et al., 2020), who were engaged in the study of different *Coptis* species in China. Their research was of great significance to the distribution and identification of *Coptis* species. In the author co-citation analysis, the document published by Japanese scholar Sato F had the highest number of citations overall. Sato F along with Morishige T, Ikezawa N, Yamada Y and other major authors formed the largest network of cited references. The second-largest co-cited network was formed by the Chinese researchers Kong WJ and Chen JH, and the Korean researcher Jung HA. Their research results are discussed below in the knowledge base section.

Sato F is an important researcher in *Coptis* researchwith him as the central author, a group of Japanese researchers has been formed (Minami et al., 2007; Ikezawa et al., 2008). Articles be Sato F have been cited numerous times, and served as the knowledge base in the *Coptis* research field. Further, Sato F is an expert in plant cell molecular biology and is among the first group of researchers worldwide to use microorganisms to successfully produce isoquinoline alkaloids (Sato et al., 2007), which are typical secondary metabolites from plants. Sato F has established cultured cells (Sato et al., 1994) that can efficiently produce isoquinoline alkaloids from plants, such as the medicinal plant *C. japonica*. He discovered a variety of enzymes that control the biosynthesis of isoquinoline alkaloids and isolated associated genes (Inui et al., 2012). Sato F’s research has made important contributions to the *Coptis* field, and his research results have been cited by many investigators in this field.

### Knowledge Base

In bibliometrics, references constitute the knowledge base of the field. We can determine the knowledge base of the *Coptis* research field by analyzing the co-cited network of references (Li et al., 2018). By combining the number of citations of a reference and the strength of the connection, three references were found that have important knowledge-based functions. The following are their main findings.

Due to the variety of *Coptis* species and because it is difficult to distinguish among them, Chen JH established a method for the identification and quantification of the main alkaloids in *C. chinensis* extracts based on ASE and UPLC. The results show that the UPLC fingerprint based on the distribution of eight major alkaloids can be used as a fast and reliable method for the identification and quality evaluation of traditional Chinese medicine (Chen et al., 2008).

Sato F used a screening method to establish a *C. japonica* cell culture capable of producing a large amount of berberine and obtained a stable and high-yield *C. japonica* cell line through repeated cloning, enabling the stable and efficient production of berberine.

Chinese researcher Kong WJ identified berberine as a new cholesterol-lowering drug (Kong et al., 2004). Clinical experiments show that berberine can reduce serum cholesterol, triglycerides, and low-density lipoprotein (LDL) cholesterol in patients with hypercholesterolemia. Animal experiments show that berberine can reduce the serum cholesterol and LDL cholesterol of hyperlipidemic hamsters and increase liver low-density lipoprotein receptor (LDLR) mRNA and LDLR protein. Using human liver cancer cells, they found that berberine upregulate the expression of LDLR depending on the activation of extracellular signal-regulated kinase (ERK). Berberine increase the expression of LDLR by stabilizing the post-transcriptional mechanism of mRNA. Using luciferase as a heterologous system for reporter genes, scholars have further identified five proximal regions of the untranslated region of LDLR mRNA 3’, which are responsible for the regulation of berberine. These findings indicate that berberine is a new lipid-lowering drug, and its mechanism of action is different from that of statins.

### Research Hotspots

A hotspot refers to a scientific issue or topic keyword that is internally related to a specific period, which is discussed in a specific set of publications, and allows the generalization of the topic in the publications. The analysis of high-frequency keywords can identify hot spots in the *Coptis* research field (Wu et al., 2019).

In this study, berberine was the keyword with the highest frequency and betweenness centrality, indicating that berberine has received widespread attention from scholars studying *Coptis*. The second-largest cluster tag in the keyword clustering, “alkaloid”, could also explain its importance. The rhizome of *Coptis* contains a variety of alkaloids, of which berberine has the highest content and is also the most widely studied. Berberine has a variety of pharmacological effects (Lee et al., 2006; Tang et al., 2009; Kabary et al., 2018) such as inhibiting pathogens, lowering hyperglycemia, inhibiting gastric ulcers, and inhibiting tumors. Its pharmacological effects of inhibiting pathogens and lowering hyperglycemia have attracted the attention of many researchers. The inhibitory activity of berberine has been evaluated against sortase, a bacterial surface protein anchoring transpeptidase, from *Staphylococcus aureus* ATCC 6538p. Berberine is a potent inhibitor of sortase, with an IC50 value of 8.7 mug/ml and has antibacterial activity against Gram-positive bacteria with a minimum inhibitory concentration (MIC) in the range of 50,400 mug/ml. These results indicate that berberine may be developed as a bacterial sortase inhibitor (Kim et al., 2004). Berberine has been prescribed for the treatment of diabetes. Glucose and lipid metabolism can be regulated by berberine via varied pathways, such as the AMP-activated protein kinase-(AMPK-) p38 MAPK-GLUT4, PI3K-Akt pathway, JNK pathway, PPARα pathway, etc. Activation of these pathways results in the up-regulation of the insulin receptor gene, thereby restoring insulin sensitivity (Yang et al., 2014). Thus, we believe that berberine has the effect of treating diabetes.

The frequency and centrality of *C. chinensis* among the keywords were second only to berberine, which was the third largest cluster in the keyword clustering. *C. chinensis* is a commonly used TCM (Wang et al., 2019). It was the most important species of *Coptis* in this field and was the main research focus of Chinese researchers, who were the largest research group in this study. The research content of *C. chinensis* mainly involved pharmacological effects and quality evaluation. Pharmacological effects of *C. chinensis* are similar to those of berberine and will not be repeated here. Quality control is a key factor in the development of the traditional medicine industry. However, a more effective quality control practice for *C. chinensis* should include the analysis of a group of major alkaloids, rather than a single species. Hence, many of the studies we included did not only involve the analysis of berberine. The UPLC-PDA method combined with ASE extraction was established to quantitatively determine the three main alkaloids berberine, palmatine and jatrorrhizine in the crude extract of *C. chinensis*. In addition, using the distribution pattern of the eight main alkaloids in the samples, the researchers established a UPLC-based fingerprinting method for the quality evaluation of *C. chinensis* (Chen et al., 2008). UPLC-based fingerprinting method provides a powerful tool for the quality evaluation of natural medicines.

In addition, this study clustered the study keywords and generated 13 clusters, of which the largest cluster was *C. japonica*. *C. japonica* was also the keyword with the strongest burst strength and the longest burst duration, which indicated that *C. japonica* occupied an important position in this field and was an active research hotspot during the evaluated period. Research evaluating *C. japonica* should be further explored. A research group focusing on *C. japonica* was formed with Japanese scholar Sato F as the central member. Their research hotspot was the use of microorganisms to produce isoquinoline alkaloids (Ikezawa et al., 2003; Yamada et al., 2011), which are metabolites of *C. japonica*. A variety of enzymes have been discovered that control the biosynthesis of isoquinoline alkaloids and related genes have been isolated, providing theoretical support for the large-scale production of berberine.

Keyword bursts can reflect that the frequency of keyword appearance has greatly changed in the short term. From the perspective of burst time, oxidative stress (OS), rat plasma and Alzheimer’s disease were the keywords with the most recent burst date, and they represent recent hotspots in *Coptis* research. OS refers to a state in which the body’s oxidation and antioxidant effects are out of balance. The state tends to be oxidized, contributing to the inflammatory infiltration of neutrophils, increased secretion of proteases, and the production of a large number of free radicals. Oxidative stress is believed to be an important factor leading to aging and disease (Friedemann et al., 2014). The most common diseases induced by elevated oxidative stress levels are heart disease, cancer, osteoarthritis, rheumatoid arthritis, diabetes, and neurodegenerative problems such as Alzheimer’s disease and Parkinson’s disease. Research related to OS suggests that Coptidis alkaloids inhibit oxidative stress, thereby improving the pathology of the above diseases (Friedemann et al., 2015). Studies involving the rat plasma concern the pharmacokinetics of *Coptis* alkaloids, which primarily measure the plasma contents of alkaloids after oral administration in rats (Liu et al., 2016). The burst of studies on Alzheimer’s disease began in 2018. The primary research indicates that Coptidis alkaloids can protect nerves and can be used in treating Alzheimer’s disease. *Coptis chinensis* Franch polysaccharide (CCP) appears to have a protective effect against amyloid-beta (Aβ)-induced toxicity in the *Caenorhabditis elegans* AD model partly by increasing lifespan, reducing Aβ accumulation, and up-regulating heat shock proteins. This study provides a new theoretical foundation regarding CCP for AD treatment (Li et al., 2018). The above subjects are recent research hotspots in the *Coptis* field, and researchers can conduct studies based on these hotspots.

## Strengths and Limitations

This study has several strengths. First, this is the first systematic analysis of *Coptis* research using scientometric methods to provide a reference source for researchers. Second, we employed two widely used scientometric software programs to simultaneously obtain our findings, both of which have been widely used in the field of scientometrics (Ke et al., 2020). However, similar to other scientometric research, our research also has some limitations. First, we only searched the WoS database, and none of the other large medical databases such as Scopus or Embase. It is worth noting that WoS is the most commonly used database in scientometric research (Lu et al., 2020). Due to the limited number of retrievable documents from the WoS, our study likely did not contain an exhaustive number of records regarding *Coptis* research, including the earliest documents. Nevertheless, the earliest document record may not be representative of the earliest *Coptis* research. In addition, the currently available bibliometric software exhibit great difficulty in analyzing data from multiple databases simultaneously. Second, all the data were extracted using software, unlike systematic reviews or data overviews that are manually extracted by two or more reviewers. Therefore, the data used to support our results may be biased. With the development of machine learning and data science, these problems may be solved in the future (Kehl et al., 2019). Third, we did not include papers related to *Coptis* in 2021 because these data were not complete when we searched the database.

## Conclusion

This study used scientometric tools to identify the knowledge base and research hotspots for *Coptis*. The documents in this field were mainly concentrated in China, Japan and South Korea. The research results of Sato F and Chen JH are widely recognized in *Coptis* field. In a study published by Sato F, he investigated both metabolic regulation and the selection of cell lines for the production of large amounts of berberine in cultured *Coptis* cells, which led to berberine being produced stably and efficiently. The Chinese scholar Chen JH pointed out that UPLC fingerprints based on the distribution of eight major alkaloids can quickly and reliably be used to perform identification verification and quality evaluation of TCM. Through document co-citation analysis, three areas were presented in the knowledge base on *Coptis* research: the identification of *Coptis* species, the production of alkaloids, and the elucidation of the efficacy of alkaloids and their mechanism of action. *Coptis* research hotspots were primarily concentrated in the three fields of berberine, *C. chinensis*, and *C. japonica*. Regarding berberine, the effects of inhibiting pathogens and lowering hyperglycemia have attracted the attention of many researchers. The research content of *C. chinensis* mainly involved pharmacological effects and quality evaluation. Using microorganisms to successfully produce isoquinoline alkaloids was the research focus of *C. japonica*. Finally, oxidative stress, rat plasma as an indicator of alkaloids content and Alzheimer’s disease emerged as the strongest burst keywords and recent hotspots related to *Coptis*. Researchers can conduct research based on the hotspots in this field.

## Data Availability

The original contributions presented in the study are included in the article/Supplementary Material, further inquiries can be directed to the corresponding author.
