# Supplementary material for: Scientometric Analysis of Medicinal and Edible Plant Coptis
Source: Front Pharmacol. 2021 Aug 12;12:725162. doi: 10.3389/fphar.2021.725162 (PMC8387930; doi:10.3389/fphar.2021.725162)
Supplement: Supplementary file 1 [file DataSheet1.docx]

Supplementary Material

# Supplementary Figures and Tables

## Supplementary Tables

Supplementary Table 1. The 13 largest clusters of keywords in *Coptis* research.

| Cluster ID | Cluster Name | Size | Silhouette | Mean(Year) | Top Terms |
| --- | --- | --- | --- | --- | --- |
| 0 | Coptis japonica | 97 | 0.859 | 2003 | Coptis japonica, expression, cell, biosynthesis, adenosyl l methionine |
| 1 | alkaloids | 76 | 0.71 | 2008 | berberine, alkaloid, rhizoma coptidi, plant, constituent |
| 2 | Coptis chinensis | 73 | 0.863 | 2008 | Coptis chinensis Franch, identification, binding, adsorption, medicinal plant |
| 3 | pharmacokinetics | 62 | 0.766 | 2009 | mechanism, ranunculaceae, palmatine, protoberberine alkaloid, jatrorrhizine |
| 4 | diabetes | 59 | 0.774 | 2014 | extract, activation, oxidative stress, antioxidant, model |
| 5 | microcalorimetry | 44 | 0.851 | 2011 | toxicity, antimicrobial activity, antioxidant activity, escherichia coli, berberine alkaloid |
| 6 | in vitro | 42 | 0.895 | 2005 | chitosan hydrogel, in vitro, rat, growth, drug, response |
| 7 | lignan | 26 | 0.952 | 1999 | flavonoid, ligan, ascorbic acid, agent, Coptis japonica makino |
| 8 | apoptosis | 26 | 0.899 | 2009 | apoptosis, extraction, breast cancer, proliferation, p53 |
| 9 | coptidis rhizoma | 23 | 0.918 | 2003 | baicalin, coptidis rhizoma, capillary electrophoresis, growth inhibition, chinensis |
| 10 | acetylcholinesterase inhibitor | 23 | 0.94 | 2002 | coptisine, performance liquid chromatography, isoquinoline alkoloid, alpha glucosidase inhibitor, acetylcholinesterase inhibitor |
| 11 | structure | 21 | 0.887 | 2009 | disease, acid, stress, microbial community, therapy |
| 12 | breeding systems | 14 | 1 | 2000 | endangered specy, conservation, allocation, diversity, fecundity |

## Supplementary Figures


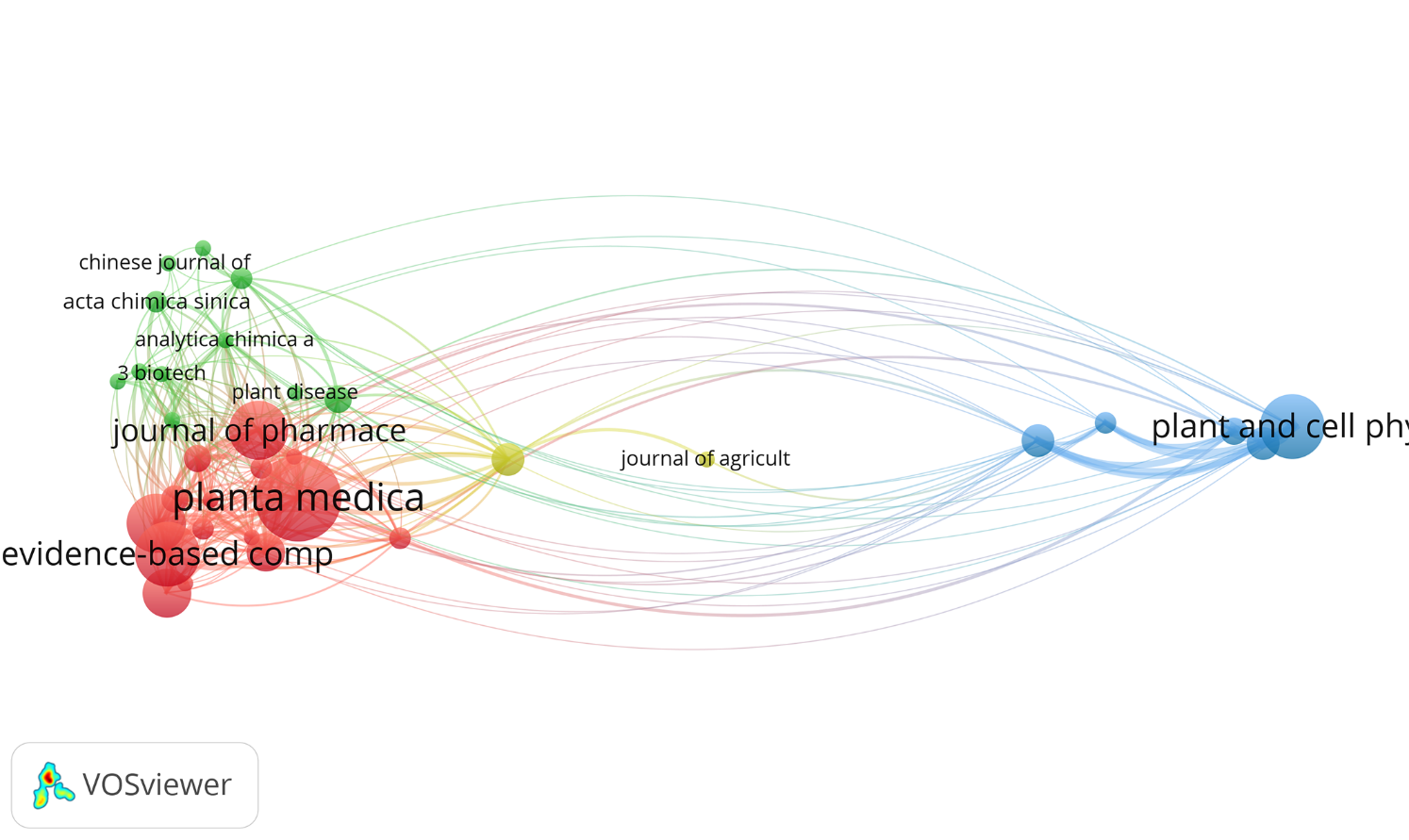


Supplementary Figure 1. Map of journal coupling analysis related to *Coptis* research.


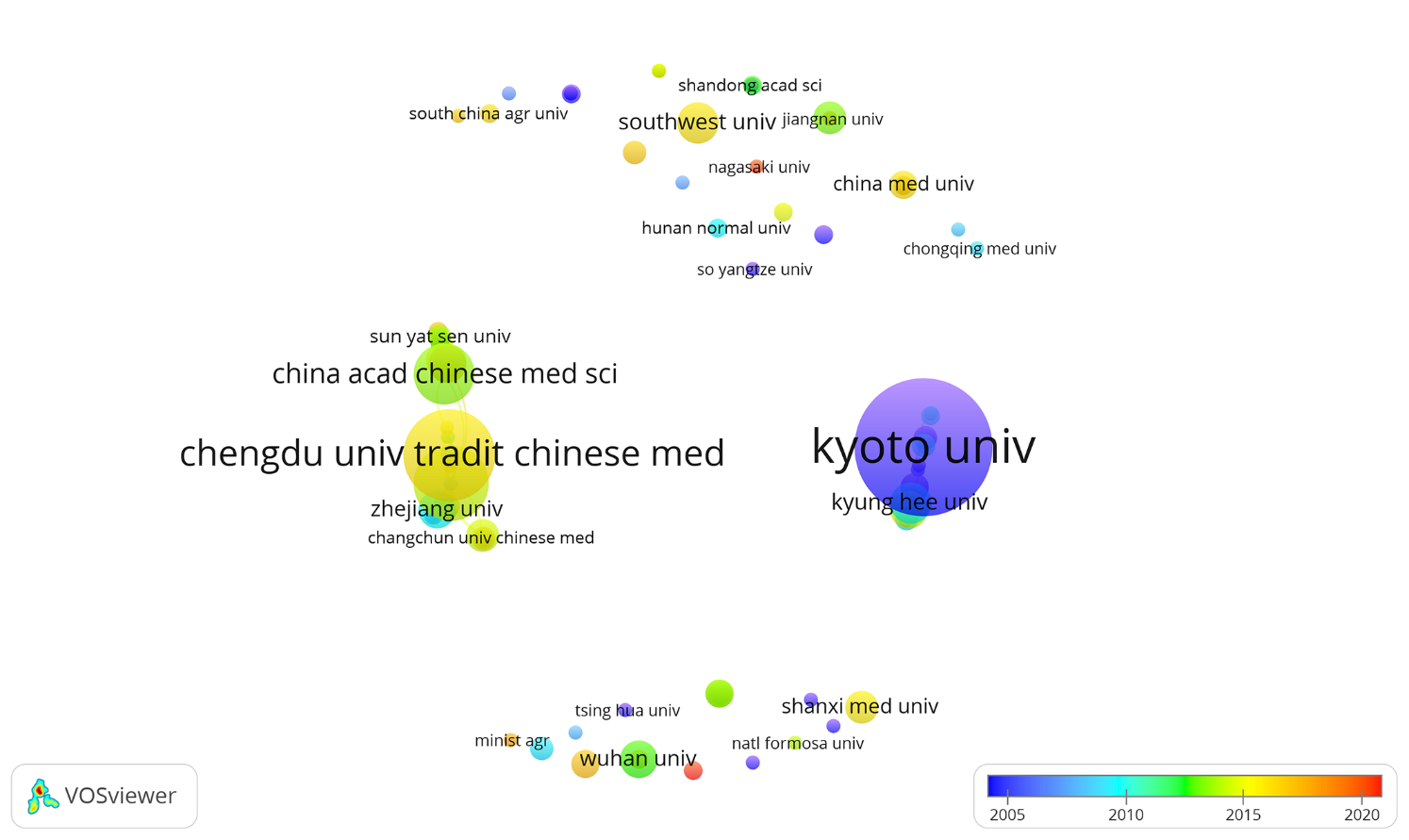


Supplementary Figure 2. Institutional collaboration network of *Coptis* research.
